# Impact of Clinical Factors on ^18^F-Flotufolastat Detection Rates in Men With Recurrent Prostate Cancer: Exploratory Analysis of the Phase 3 SPOTLIGHT Study

**DOI:** 10.1016/j.adro.2024.101532

**Published:** 2024-05-01

**Authors:** Benjamin H. Lowentritt, Ashesh B. Jani, Brian T. Helfand, Edward M. Uchio, Michael A. Morris, Jeff M. Michalski, Albert Chau, Phillip Davis, Brian F. Chapin, David M. Schuster, SPOTLIGHT Study Group

**Affiliations:** a Chesapeake Urology Research Associates, Towson, Maryland; b Department of Radiation Oncology, Winship Cancer Institute of Emory University, Atlanta, Georgia; c NorthShore University Health System, Evanston, Illinois; d University of California Irvine Medical Center, Irvine, California; e Advanced Molecular Imaging and Therapy, Glen Burnie, Maryland; f Department of Radiation Oncology, Washington University School of Medicine, St Louis, Missouri; g Blue Earth Diagnostics Ltd, Oxford, United Kingdom; h Blue Earth Diagnostics, Monroe Township, New Jersey; i Department of Urology, The University of Texas MD Anderson Cancer Center, Houston, Texas; j Division of Nuclear Medicine and Molecular Imaging, Department of Radiology and Imaging Sciences, Emory University, Atlanta, Georgia

## Abstract

**Purpose:**

^18^F-Flotufolastat (^18^F-rhPSMA-7.3) is a newly approved prostate-specific membrane antigen targeting radiopharmaceutical for diagnostic imaging of prostate cancer (PCa). SPOTLIGHT (National Clinical Trials 04186845) evaluated ^18^F-flotufolastat in men with suspected PCa recurrence. Here, we present results of predefined exploratory endpoints from SPOTLIGHT to evaluate the impact of clinical factors on ^18^F-flotufolastat detection rates (DR).

**Methods and Materials:**

The impact of baseline prostate-specific antigen (PSA), PSA doubling time (PSAdt), and International Society of Urologic Pathology Grade Group (GG) on ^18^F-flotufolastat DR was evaluated among all SPOTLIGHT patients with an evaluable scan, with DR stratified according to the patients’ prior treatment (radical prostatectomy ± radiation therapy [RP] or radiation therapy only [RT]). The patients underwent positron emission tomography 50 to 70 minutes after receiving ^18^F-flotufolastat (296 MBq IV), and scans were read by 3 blinded central readers, with the majority read representing agreement between ≥2 readers.

**Results:**

In total, 389 men (median PSA: 1.10 ng/mL) were evaluable. By majority read, ^18^F-flotufolastat identified distant lesions in 39% and 43% of patients treated with prior RP or RT, respectively. The overall DR broadly increased with increasing PSA (<0.2 ng/mL: 33%; ≥10 ng/mL: 100%). Among patients with PSA <1 ng/mL, 68% had positive scans, and 27% had extrapelvic findings. PSAdt was available for 145/389 (37%) patients. PSAdt did not appear to influence ^18^F-flotufolastat DR (77%-90% across all PSAdt categories). Among patients with prior RP, DR ranged from 70% to 83% across PSAdt categories, and 100% DR was reported for all post-RT patients. In total, 362/389 (93%) patients had baseline GG data. Overall DRs were uniformly high (75%‒95%) across all GG. When stratified by prior treatment, DRs across all GG were 69% to 89% in patients with prior RP and ≥96% in patients with prior RT.

**Conclusions:**

^18^F-Flotufolastat-positron emission tomography enabled the accurate detection of recurrent PCa lesions across a wide range of PSA, PSAdt, and International Society of Urologic Pathology GG, thus supporting its clinical utility for a broad range of patients with recurrent PCa.

## Introduction

Accurately determining the location and extent of recurrent prostate cancer lesions with a sensitive imaging modality that allows their early detection may facilitate optimal decision- making for the management of patients with biochemical recurrence (BCR), as indicated by rising prostate-specific antigen (PSA) levels. Specifically, the identification of distant disease at the time of consideration of salvage radiation therapy (RT) would allow physicians and patients to opt for systemic or metastases-directed therapy, thus foregoing unnecessary curative salvage radiation treatment that would ultimately be futile and sparing patients from potential radiation-induced side effects.^1^^,^^2^

Similarly, confidently confirming the absence of disease with a sensitive imaging modality in patients with rising PSA may help support the decision to delay initiation of systemic therapy (androgen deprivation therapy/novel hormone therapy, chemotherapy, or both), which is also associated with significant side effects.^3^, ^4^, ^5^

^18^F-Flotufolastat (^18^F-rhPSMA-7.3) is a novel, next-generation, high-affinity prostate-specific membrane antigen (PSMA)-targeting radiopharmaceutical that has recently been approved by the Food and Drug Administration for positron emission tomography (PET) of PSMA-positive lesions in men with suspected metastasis who are candidates for initial definitive therapy or for men with suspected BCR based on serum PSA level.^6^^,^^7^ Early clinical data showed ^18^F-flotufolastat to have lower average urinary excretion than reported values for other renally cleared PSMA-PET radiopharmaceuticals,^8^ and a recent post hoc analysis of 2 phase 3 clinical trials confirmed that the urinary excretion of ^18^F-flotufolastat does not impact image assessment for the majority of patients.^9^

The phase 3 SPOTLIGHT study (National Clinical Trials 04186845) evaluated the diagnostic accuracy of ^18^F-flotufolastat in men who developed BCR after prior curative-intent treatment of prostate cancer.^10^ The primary endpoints of SPOTLIGHT have been reported previously and show ^18^F-flotufolastat to have a clinically meaningful diagnostic performance in patients with BCR of prostate cancer, with a verified detection rate (VDR) of 57%.^10^

It is well known that certain clinical factors, such as PSA levels, PSA kinetics (eg, PSA doubling time [PSAdt]), and Gleason scores, can influence the diagnostic performance of some PET radiopharmaceuticals, including ^11^C- or ^18^F-choline and ^18^F-fluciclovine.^11^, ^12^, ^13^, ^14^ This is likely because higher baseline PSA and Gleason scores and shorter PSAdt are a reflection of disease aggressiveness and burden and have been recognized as independent prognostic factors for prostate cancer.^15^^,^^16^

Data for other PSMA-targeting radiopharmaceuticals such as ^68^Ga-PSMA-11 and ^18^F-piflufolastat (^18^F-DCFPyL) have also reported an association between increasing PSA levels, Gleason scores, and DR in patients with BCR.^17^^,^^18^ The diagnostic utility of PSMA-PET in patients with BCR is being increasingly recognized,^19^ leading to its inclusion in the latest prostate cancer guidelines.^7^^,^^12^

Here, we present results of predefined exploratory efficacy endpoints from the SPOTLIGHT study to evaluate the impact of clinical factors such as baseline PSA, PSAdt, and International Society of Urologic Pathology (ISUP) Grade Group^20^ on the ^18^F-flotufolastat DR in patients with suspected BCR.

## Methods and Materials

### Study design and patients

SPOTLIGHT was a phase 3, prospective, multicenter, open-label, single-arm study conducted in accordance with the Declaration of Helsinki and the International Council on Harmonization Guidelines for Good Clinical Practice. The study protocol was approved by each study site's independent ethics committee, and all patients provided written informed consent.

The full methods of SPOTLIGHT have been reported previously,^10^ but in brief, men (>18 years) with elevated PSA suspicious for recurrence of previously treated, localized prostate adenocarcinoma were eligible for inclusion if they were being considered for curative-intent salvage therapy.

An elevated PSA was defined as ≥0.2 ng/mL with a subsequent confirmatory value of ≥0.2 ng/mL for patients previously treated with radical prostatectomy (RP) ± adjuvant therapy or as nadir +2 ng/mL for patients treated with prior RT, brachytherapy, or focal gland therapy. Patients were required to have discontinued androgen deprivation therapy ≥16 weeks before screening.^10^

The patients received 8 mCi (296 MBq) ± 20% ^18^F-flotufolastat, administered as an intravenous bolus injection, and PET/computed tomography (CT) was conducted 50 to 70 min later.^10^

Images were read by 3 trained, independent central readers who were blinded to all clinical information.^10^ The readers considered a lesion suspicious if ^18^F-flotufolastat uptake was greater than physiological uptake in that tissue or greater than adjacent background (where no physiological uptake was expected).^10^

For this predefined exploratory analysis, the impact of baseline PSA levels, PSAdt, and ISUP Grade Group on the overall patient- and region-level DR (defined as the number of patients with ≥1 PET-positive lesion, divided by the number of patients who had an evaluable PET/CT scan) was evaluated in the evaluable PET scan population (EPSP; ie, all patients who received an ^18^F-flotufolastat injection and underwent PET/CT). Results are further stratified according to patients’ prior treatment (RP ± RT vs RT only).

Patient demographics and baseline characteristics were recorded at screening. PSAdt was calculated by doing a regression of historical natural log PSA on the date of measurement and dividing natural log 2 (0.693) by the slope, using the last 3 values in the 2 years before ^18^F-flotufolastat administration (1 month was assumed to be 30.5 days). In cases with <3 acceptable measurements in the previous 2 years, PSAdt was considered missing. PSAdt was categorized as <6, ≥6 to <12, ≥12 to <24, ≥24 months, or not estimable (for nonchanging and decreasing PSA [slope ≤0]).

DR data are summarized here as point estimates (percentages) for the majority read (agreement between ≥2 readers), alongside the corresponding 2-sided, exact 95% CI. The study was not designed to compare the different subgroups and therefore no hypothesis has been set a priori for intergroup comparisons and no formal analyses for statistical differences were performed.

## Results

### Patients

In total, 420 patients were enrolled across 27 sites (24 in the United States, 3 in Europe) between May and December 2020 (Fig. E1). Of these, 389 had an evaluable ^18^F-flotufolastat scan and comprised the EPSP.

Baseline demographics and disease characteristics are summarized in Table 1. Mean age was 68 years, and majority of patients (60%) had a Gleason score of 7. Median baseline PSA was 1.10 ng/mL. In total, 305/389 (78%) patients (median [range] PSA, 0.68 [0.09-32.20] ng/mL) had previously undergone RP, and 76/389 (20%) (median [range] PSA, 4.41 [0.03-134.60] ng/mL) had previously been treated with RT only.

**Table 1:** Baseline characteristics for the evaluable PET scan population

	EPSP (N = 389)
Age, years	
Mean	68.3
SD	7.93
Range	43-86
Gleason score, n (%)	
≤6	39 (10%)
7	232 (60%)
≥8	103 (26%)
Missing	15 (3.9%)
ISUP grade group, n (%)	
1	39 (10%)
2	104 (27%)
3	116 (30%)
4	40 (10%)
5	63 (16%)
Missing	27 (6.9%)
Time from initial prostate cancer diagnosis, months	
Median	69
Range	2-409
Prior therapy, n (%)	
With prior prostatectomy	305 (79%)
- With radiation therapy	137 (45%)
- Without radiation therapy	168 (55%)
Without prior prostatectomy	84 (22%)
- Radiation therapy	76 (90%)
- Other therapy	7 (8.3%)
- No other therapy	1 (1.2%)
Baseline PSA, ng/mL	
Median	1.10
Range	0.03-134.6
PSA <0.5, n (%)	121 (31%)
PSA ≥0.5 ‒ <1.0, n (%)	67 (17%)
PSA ≥1.0 ‒ <2.0, n (%)	45 (12%)
PSA ≥2.0 ‒ <5.0, n (%)	88 (23%)
PSA ≥5.0 ‒ <10.0, n (%)	36 (9%)
PSA ≥10.0, n (%)	32 (8%)

*Abbreviations:* EPSP = evaluable PET scan population; ISUP = International Society of Urologic Pathology; PET = positron emission tomography; PSA = prostate-specific antigen.

### Overall and regional ^18^F-flotufolastat DR stratified by patients’ prior treatment

**Figure 1:**
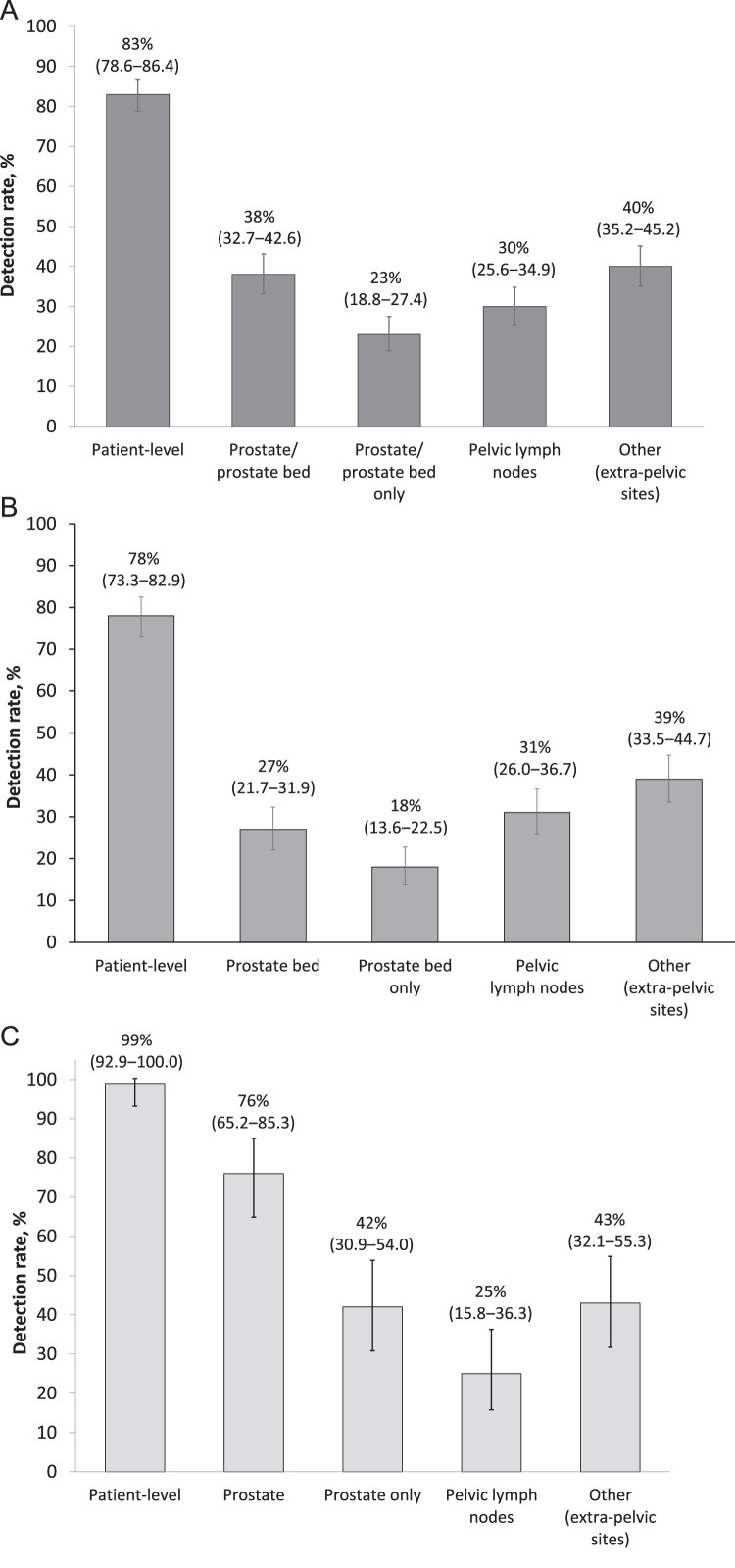
(A) Overall (patient-level) and regional ^18^F-flotufolastat detection rates in patients with suspected biochemical recurrence. (B) ^18^F-Flotufolastat detection rates in patients treated with prior radical prostatectomy ± radiation therapy or (C) prior radiation therapy only.

Figure 1A presents the overall DR (by majority read) at patient-level and by region. In the 389 patients with an evaluable ^18^F-flotufolastat-PET, the overall DR was 83% (322/389; 95% CI, 78.6%-86.4%). In the prostate/bed, the DR was 38% (146/389; 95% CI, 32.7%-42.6%); 23% of patients (89/389; 95% CI,18.8%-27.4%) had positive findings confined to the prostate/bed. In pelvic lymph nodes, the DR was 30% (117/389; 95% CI, 25.6%-34.9%), and in other (extrapelvic) sites, the DR was 40% (156/389; 95% CI, 35.2%-45.2%).

Figure 1B and 1C present the overall and regional DR (by majority read) for patients treated with prior RP or with prior RT only. Overall (patient-level) DR in patients treated with prior RP was 78% (239/305; 95% CI, 73.3%-82.9%; Fig. 1B). In the prostate bed, DR was 27% (81/305; 95% CI, 21.7%-31.9%), with 18% of this subgroup (54/305; 95% CI, 13.6%-22.5%) having ^18^F-flotufolastat-positive lesions in the prostate bed only. DR was 31% (95/305; 95% CI, 26.0%-36.7%) in pelvic lymph nodes and 39% (119/305; 95% CI, 33.5%-44.7%) in other (extrapelvic) sites (Fig. 1B).

In total, 75/76 (99%; 95% CI, 92.9%-100.0%) of patients with prior RT had a positive ^18^F-flotufolastat scan (Fig. 1C). Most of these patients had positive findings in the prostate (58/76; 76%; 95% CI, 65.2-85.3), but only 32/76 (42%; 95% CI, 30.9-54.0) had positive findings confined to the prostate. ^18^F-Flotufolastat-avid pelvic lymph nodes were detected in 25% (19/76; 95% CI, 15.8-36.3) of patients with prior RT and, notably, extrapelvic lesions (eg, “other”) were detected in 43% (33/76; 95% CI, 32.1-55.3) (Fig. 1C).

### Impact of baseline PSA levels on ^18^F-flotufolastat DR

**Figure 2:**
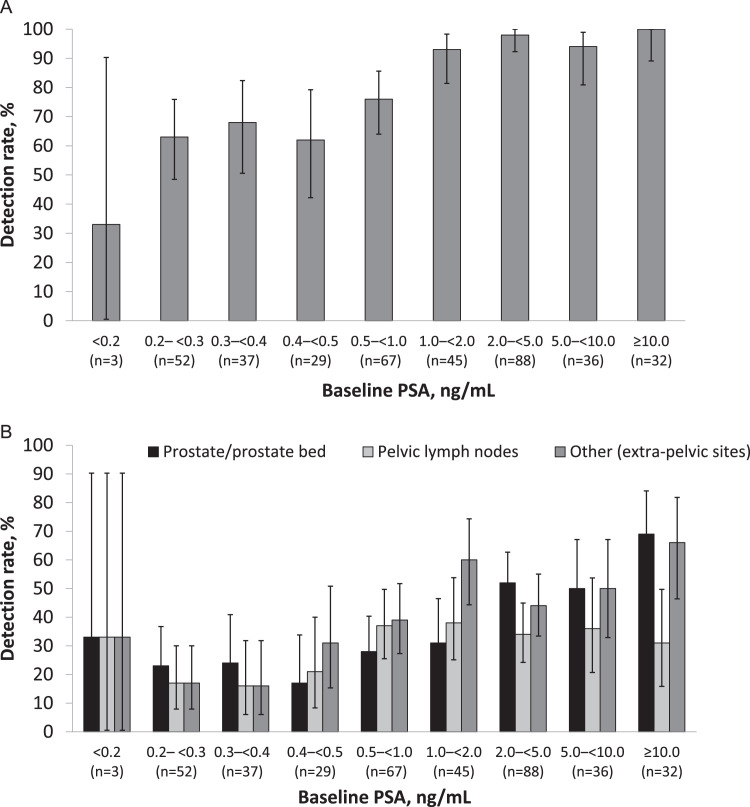
(A) Patient-level and (B) region-level ^18^F-flotufolastat detection rates (majority read) stratified by baseline prostate-specific antigen.

We examined the impact of baseline PSA levels on ^18^F-flotufolastat DR in all patients who underwent ^18^F-flotufolastat-PET (EPSP). Figure 2 shows the majority read data, stratified by baseline PSA levels; full patient- and region-level DR are provided in Table E1.

Moderate to high DR were observed across most PSA categories. The overall (patient-level) DR broadly increased with increasing PSA and ranged from 33% (1/3; 95% CI, 0.8%-90.6%) at PSA <0.2 ng/mL to 100% (32/32; 95% CI, 89.1%-100.0%) at PSA ≥10 ng/mL (Fig. 2A). In total, 64% (77/121) of patients with a PSA <0.5 ng/mL and 68% (128/188) of patients with a PSA <1 ng/mL had a positive ^18^F-flotufolastat scan by majority read (Fig. 2A).

In the prostate/prostate bed, DR also broadly increased in line with PSA levels, with values ranging from 22% (95% CI, 15.2%-30.8%) at PSA <0.5 ng/mL to 69% (95% CI, 50.0%-83.9%) at PSA ≥10 ng/mL (Fig. 2B).

Detection in pelvic lymph nodes was more consistent across the PSA categories. The DR was lowest for patients with PSA levels <0.5 ng/mL (18%; 95% CI, 11.8%-26.2%), but it was consistently higher across PSA levels from 0.5 to 1.0 ng/mL (37%; 95% CI, 25.8%-50.0%) to ≥10 ng/mL (31%; 95% CI, 16.1%-50.0%; Fig. 2B).

For other (extrapelvic) sites, DR broadly increased with rising PSA levels, with values ranging from 21% (95% CI, 13.8%-29.0%) at PSA <0.5 ng/mL to 66% (95% CI, 46.8%-81.4%) at PSA ≥10 ng/mL (Fig. 2B).

For patients with PSA <0.2 ng/mL, DR was 33% in each of the 3 regions (95% CI for all, 0.8%-90.6%), although there were limited numbers of patients in this category.

Overall, regional DRs with ^18^F-flotufolastat were broadly consistent across all low-PSA categories below 0.5 ng/mL. Of note, extrapelvic lesions were observed in 21% (25/121; 95% CI, 13.8%-29.0%) of patients with a PSA <0.5 ng/mL and in 27% (51/188; 95% CI, 20.9%-34.1%) of all patients with PSA <1 ng/mL (Fig. 2B, Table E1).

**Figure 3:**
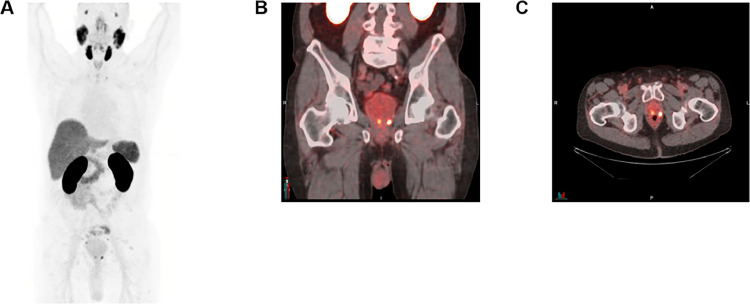
Representative ^18^F-flotufolastat-positron emission tomography (PET) images. Maximum intensity projection (A) and fused ^18^F-flotufolasta-PET/computed tomography images (B,C) of a 71-year-old patient with biochemical recurrence after radical prostatectomy (baseline prostate-specific antigen: 0.1 ng/mL; prostate-specific antigen doubling time: 30.7 months). ^18^F-Flotufolastat-avid lesions were identified in the prostate bed (left seminal vesicle) and in pelvic lymph nodes (B,C), which were subsequently confirmed as true positive by imaging standard of truth (^18^F-fluciclovine-PET).

Representative images from a patient with low baseline PSA (<0.1 ng/mL) are shown in Figure 3.

### ^18^F-Flotufolastat DR stratified by PSAdt

**Figure 4:**
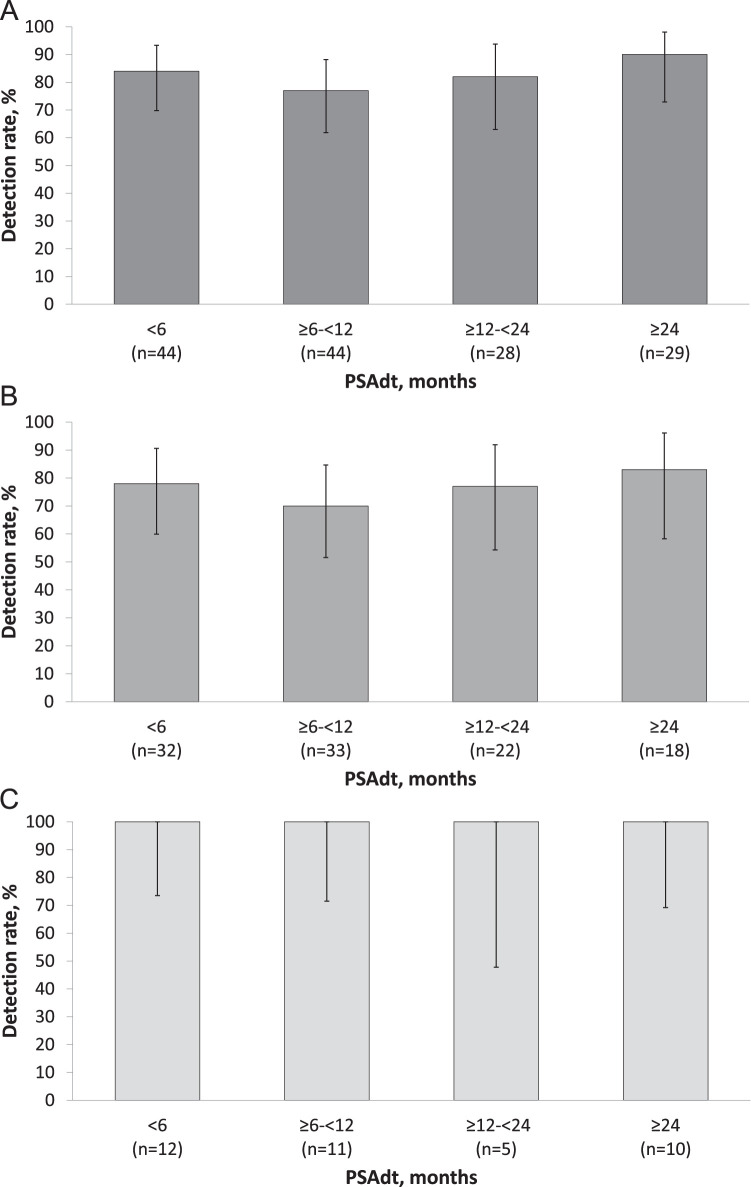
^18^F-Flotufolastat detection rates (majority read) by prostate-specific antigen doubling time in (A) all patients with prostate-specific antigen doubling time data available (n = 145) and stratified by prior treatment; (B) prior radical prostatectomy ± radiation therapy (n = 105); and (C) prior radiation therapy only (n = 38).

PSAdt did not appear to influence the ^18^F-flotufolastat DR among the 145/389 (37%) patients for whom PSAdt could be determined; overall (patient-level) DR by majority read were 84% (37/44; 95% CI, 69.9%-93.4%), 77% (34/44; 95% CI, 62.2%-88.5%), 82% (23/28; 95% CI, 63.1%-93.9%), and 90% (26/29; 95% CI, 72.6%-97.8%) across PSAdt categories <6, ≥6 to <12, ≥12 to <24, and ≥24 months, respectively (Fig. 4A).

There was also no clear association between PSAdt and DR by region, irrespective of prior treatment (Table E2).

Among patients previously treated with RP, DR by majority read were 78% (25/32; 95% CI, 60.0%-90.7%), 70% (23/33; 95% CI, 51.3%-84.4%), 77% (17/22; 95% CI, 54.6%-92.2%), and 83% (15/18; 95% CI, 58.6%-96.4%) for PSAdt of <6, ≥6 to <12, ≥12 to <24, and ≥24 months, respectively (Fig. 4B). All patients with prior RT had a positive scan (100% DR) irrespective of PSAdt (Fig. 4C).

### ^18^F-Flotufolastat DR stratified by ISUP Grade Group

**Figure 5:**
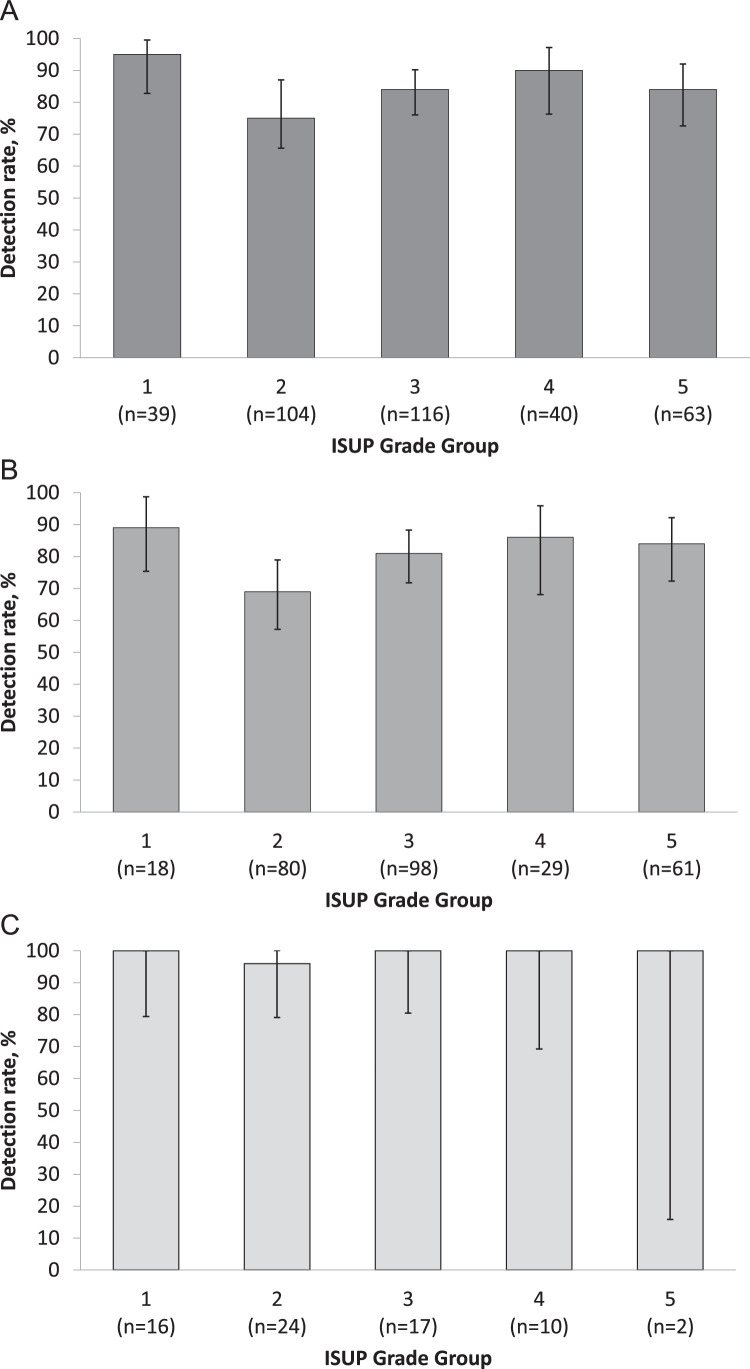
^18^F-Flotufolastat detection rates (majority read) by International Society of Urologic Pathology Grade Group in (A) all patients with available data (n = 362) and stratified by previous treatment; (B) prior radical prostatectomy ± radiation therapy (n = 286); and (C) radiation therapy only (n = 69).

In patients for whom baseline ISUP Grade Group data were available (362/389; 93%), overall DR (majority read) were uniformly high, ranging from 75% to 95% across all ISUP Grade Groups (Fig. 5A). In patients with prior RP the DR were 89% (16/18; 95% CI, 65.3%-98.6%), 69% (55/80; 95% CI, 57.4%-78.7%), 81% (79/98; 95% CI, 71.4%-87.9%), 86% (25/29; 95% CI, 68.3%-96.1%), and 84% (51/61; 95% CI, 71.9%-91.8%), for Grade Groups 1, 2, 3, 4, and 5, respectively (Fig. 5B). In patients with prior RT, DRs were 100% (16/16; 95% CI, 79.4%-100%), 96% (23/24; 95% CI, 78.9%-99.9%), 100% (17/17; 95% CI, 80.5%-100%), 100% (10/10; 95% CI, 69.2%-100%), and 100% (2/2; 95% CI, 15.8%-100%) for Grade Groups 1, 2, 3, 4, and 5, respectively (Fig. 5C).

There was a trend toward higher DR in the prostate/bed at lower Grade Groups and higher DR in pelvic lymph node and extrapelvic sites at higher Grade Groups, irrespective of prior treatment, although low numbers of patients in some of the ISUP categories limit meaningful conclusions (Table E2).

## Discussion

Overall DR (without verification of imaging findings) still represents one of the main endpoints traditionally reported in diagnostic imaging studies of prostate cancer. Here, we report exploratory endpoint data from SPOTLIGHT to assess the overall patient- and region-level DR in relation to various clinical parameters such as baseline PSA level, PSAdt, and ISUP Grade Group, as well as previous treatment. This analysis is of relevance because these factors, particularly PSA levels, are known to influence the DR of PET radiopharmaceuticals,^11^, ^12^, ^13^, ^14^ likely as a reflection of disease burden.

Our data show that, among all evaluable patients, the overall DR of ^18^F-flotufolastat by majority read was high (83%). DRs by region were similarly distributed in the prostate/prostate bed and other extrapelvic sites (38%-40%), and DR in pelvic lymph nodes was slightly lower (30%). DR remained consistently high when patients were stratified by prior treatment, especially in patients treated with prior RT, who reported DR of 99% compared with 78% in patients treated with prior RP, although this higher DR among post-RT patients is likely a reflection of their higher median PSA levels. Importantly, 39% and 43% of patients treated with prior RP or RT, respectively, had positive ^18^F-flotufolastat scans in distant (extrapelvic) sites. It must be noted, however, that these DR are unverified and include both true-positive and any false-positive results.

As could be expected, overall (patient-level) DR in our study broadly increased with increasing PSA, and a similar pattern was observed in the prostate/bed and in extrapelvic sites when DRs were analyzed by region. Importantly, DRs with ^18^F-flotufolastat-PET were high across a broad range of PSA categories, indicating that this is a highly sensitive imaging technique even at very low PSA values (<0.5 ng/mL), where almost two-thirds of patients were found to have positive scans, and a fifth of patients had extrapelvic lesions. The DR of ^18^F-flotufolastat-PET in patients with PSA <0.5 ng/mL (64%) compares favorably with DR of ^18^F-piflufolastat and ^68^Ga-PSMA-11 in a similar population (36% and 38%, respectively).^21^^,^^22^

In this analysis, 3 patients had a PSA <0.2 ng/mL, which is below the threshold of the American Society for Radiation Oncology/American Urologic Association definition of BCR (2 consecutive rises in PSA ≥0.2 ng/mL)^23^ as well as the European Association of Urology (EAU)-European Society for Radiotherapy and Oncology (ESTRO)-International Society of Geriatric Oncology (SIOG) definition (PSA level >0.4 ng/mL and rising after RP).^12^ One patient with PSA <0.2 ng/mL was found to have a positive ^18^F-flotufolastat-PET scan, again highlighting the potential clinical utility of this test in patients with very low PSA levels, although this needs confirmation in a larger group of patients.

Previous studies with ^68^Ga-PSMA-11-PET and ^18^F-piflufolastat in recurrent prostate cancer have frequently reported a clear association between positive PET scans and various factors such as baseline PSA, PSAdt, Gleason score, and prior treatment.^17^^,^^18^^,^^24^ However, other studies have found that DRs do not always correlate with PSAdt or Gleason score.^25^, ^26^, ^27^ In our study, the overall DR with ^18^F-flotufolastat-PET increased with baseline PSA levels and was uniformly high across all PSAdt and ISUP Grade Groups regardless of prior treatment, although there were low numbers of patients in some categories.

Of note, the ability of ^18^F-flotufolastat-PET to detect distant lesions in a significant proportion of patients with BCR is clinically relevant, as the presence of metastases is likely to lead to changes in management, especially in cases where salvage local therapy in postradiation patients is being considered, as this procedure alone would be futile in patients with potentially metastatic disease. The value of ^18^F-flotufolastat-PET in this setting is concordant with other studies of ^68^Ga-PSMA-11 and ^18^F-piflufolastat, which have also reported changes in management in a high proportion of patients with BCR and equivocal conventional imaging after undergoing ^68^Ga-PSMA-11-PET or ^18^F-piflufolastat-PET to clarify equivocal lesions.^28^, ^29^, ^30^, ^31^, ^32^

Limitations of our study include the fact that 7% of patients had missing data for the ISUP Grade Group, and only 37% of patients had sufficient data to robustly determine a PSAdt. Although ^18^F-flotufolastat DR remained consistently high across all PSAdt and ISUP Grade Group categories*,* irrespective of prior treatment, this exploratory analysis was not powered to make any correlations or intergroup comparisons. In addition, the study did not include an active comparator and, in the absence of head-to-head studies of ^18^F-flotufolastat-PET with other PSMA-PET agents, drawing any comparisons between the performance of these agents should be done with caution because of the potential impact of different patient populations, endpoints, scanning methods, and individual reader performance on imaging outcomes. Lastly, although the data here report the high overall DR for ^18^F-flotufolastat-PET, these do not represent verified findings. However, the previous report of the SPOTLIGHT primary endpoint data show that among 366 of the patients evaluated who had standard-of-truth data (either histopathology [n = 69] or confirmatory imaging [n = 297]), the majority read VDR was 57% (208/366; 95% CI, 51.6-62.0), exceeding the prespecified statistical threshold, with an even greater VDR observed among the subset with a histopathology standard of truth (81% [56/69]; 95% CI, 69.9-89.6).^10^

Nonetheless, the encouraging DRs reported here with ^18^F-flotufolastat-PET across a wide range of PSA levels and ISUP Grade Groups irrespective of prior treatments are likely because of its favorable biodistribution profile, with sustained plasma bioavailability, limited urinary bladder activity at the point of imaging, and high-affinity receptor binding and internalization compared with other radiopharmaceuticals.^8^^,^^9^^,^^33^^,^^34^ Such properties may offer beneficial diagnostic advantages and likely contributed to the high DR reported with ^18^F-flotufolastat-PET, even in patients with very low PSA levels.

## Conclusion

High DRs with ^18^F-flotufolastat were observed over a wide range of baseline PSA values, PSAdt categories, and ISUP Grade Groups, regardless of prior treatment. These results indicate that ^18^F-flotufolastat-PET may be a useful tool for treatment planning in a broad range of patients with early biochemical recurrence of prostate cancer where curative salvage therapy is of prime consideration.

## Disclosures

Benjamin H. Lowentritt, none. Ashesh B. Jani has received speaker's honoraria from Blue Earth Diagnostics Ltd. Brian T. Helfand has acted as a study investigator for Blue Earth Diagnostics Ltd, but has no other conflicts to disclose. Edward M. Uchio reports a clinical trial contract with Blue Earth Diagnostics Ltd. Michael A. Morris has participated in advisory boards for Oranomed, RayzeBio, and Fusion, has a leadership or fiduciary role for AMIT, ACNM, and SNMMI, stock options for AMIT, Gentem Health, and SoftThread, and reports a US Patent application for a radioactive patient-delivery system. Jeff M. Michalski, none. Albert Chau received consultancy fees from Blue Earth Diagnostics Ltd for data management and statistical services. Phillip Davis is an employee of Blue Earth Diagnostics Inc. Brian F. Chapin, none. David M. Schuster has acted as a consultant for Global Medical Solutions Taiwan, Progenics Pharmaceuticals, Inc, Heidelberg University, and DuChemBio Co Ltd. He participates through the Emory Office of Sponsored Projects in full compliance with Emory University–sponsored research and conflict of interest regulations in sponsored grants including those funded or partially funded by Blue Earth Diagnostics, Ltd, Nihon MediPhysics Co, Ltd, Telix Pharmaceuticals (US) Inc, Advanced Accelerator Applications, FUJIFILM Pharmaceuticals U.S.A., Inc, and Amgen Inc. He participates in educational initiatives with School of Breast Oncology, PrecisCa and provides medicolegal consulting vetted through Emory SOM.

## Appendix Supplementary materials

SUPPLEMENTARY MATERIALS Image, application 1

## Footnotes

Supplementary material associated with this article can be found in the online version at doi:10.1016/j.adro.2024.101532.
