# Supplementary material for: Impact of Clinical Factors on 18F-Flotufolastat Detection Rates in Men With Recurrent Prostate Cancer: Exploratory Analysis of the Phase 3 SPOTLIGHT Study
Source: Adv Radiat Oncol. 2024 May 1;9(8):101532. doi: 10.1016/j.adro.2024.101532 (PMC11298587; doi:10.1016/j.adro.2024.101532)
Supplement: SUPPLEMENTARY MATERIALS [file mmc1.pdf]

SUPPLEMENTARY MATERIALS

Supplementary Figure 1. Standards for Reporting of Diagnostic Accuracy (STARD) flow diagram of SPOTLIGHT study participants.

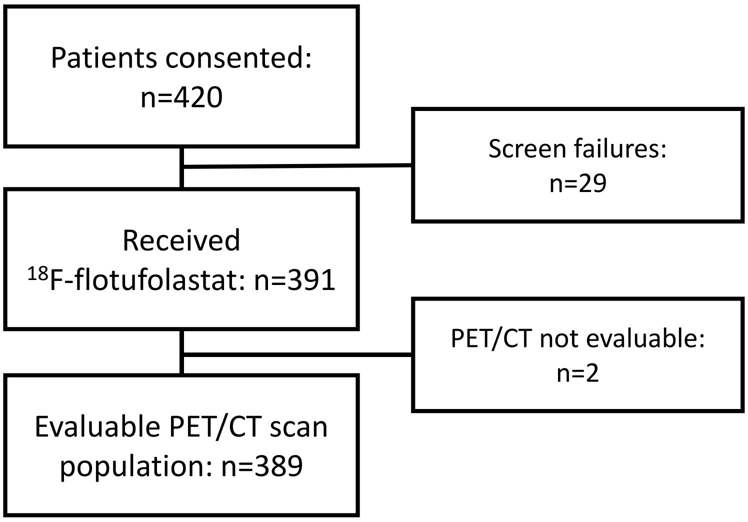

CT, computed tomography; PET, positron emission tomography.

Supplementary Table 1. Patient- and region-level ^{18}F-flotufolastat DR stratified by baseline PSA levels in patients with suspected biochemical recurrence of prostate cancer.

DR, n (%)	Baseline PSA (ng/mL)								
	**<0.2**	**0.2–<0.3**	**0.3–<0.4**	**0.4–<0.5**	**0.5–<1.0**	**1–<2.0**	**2.0–<5.0**	**5.0–<10.0**	**≥10.0**
**All patients**	**n = 3**	**n = 52**	**n = 37**	**n = 29**	**n = 67**	**n = 45**	**n = 88**	**n = 36**	**n = 32**
**Patient-level DR**	1 (33%)	33 (64%)	25 (68%)	18 (62%)	51 (76%)	42 (93%)	86 (98%)	34 (94%)	32 (100%)
**DR by region**									
**Prostate/bed**	1 (33%)	12 (23%)	9 (24%)	5 (17%)	19 (28%)	14 (31%)	46 (52%)	18 (50%)	22 (69%)
**PLN**	1 (33%)	9 (17%)	6 (16%)	6 (21%)	25 (37%)	17 (38%)	30 (34%)	13 (36%)	10 (31%)
**Other sites**	1 (33%)	9 (17%)	6 (16%)	9 (31%)	26 (39%)	27 (60%)	39 (44%)	18 (50%)	21 (66%)

DR, detection rate; PLN, pelvic lymph nodes; PSA, prostate-specific antigen.

Supplementary Table 2. Region-level ^{18}F-flotufolastat detection rates stratified by PSA doubling time and ISUP Grade Group.

DR, n (%)	PSAdt (months)				ISUP Grade Group				
	**<6**	**6–<12**	**12–<24**	**≥24**	**1**	**2**	**3**	**4**	**5**
**All patients**	37/44 (84%)	34/44 (77%)	23/28 (82%)	26/29 (90%)	37/39 (95%)	78/104 (75%)	97/116 (84%)	36/40 (90%)	53/63 (84%)
**Prostate/bed**	13 (30%)	18 (41%)	10 (36%)	14 (48%)	32 (82%)	47 (45%)	33 (28%)	14 (35%)	12 (19%)
**PLN**	11 (25%)	10 (23%)	10 (36%)	7 (24%)	6 (15%)	23 (22%)	42 (36%)	12 (30%)	27 (43%)
**Other sites**	22 (50%)	15 (34%)	9 (32%)	12 (41%)	7 (18%)	26 (25%)	52 (45%)	20 (50%)	38 (60%)
**Prior RP**	25/32 (78%)	23/33 (70%)	17/22 (77%)	15/18 (83%)	16/18 (89%)	55/80 (69%)	79/98 (81%)	25/29 (86%)	51/61 (84%)
**Prostate bed**	5 (16%)	13 (39%)	6 (27%)	4 (22%)	13 (72%)	27 (34%)	22 (22%)	5 (17%)	11 (18%)
**PLN**	7 (22%)	6 (18%)	8 (36%)	5 (28%)	2 (11%)	19 (24%)	34 (35%)	10 (35%)	26 (43%)
**Other sites**	14 (44%)	10 (30%)	7 (32%)	8 (44%)	4 (22%)	17 (21%)	41 (42%)	13 (45%)	36 (59%)
**Prior RT**	12/12 (100%)	11/11 (100%)	5/5 (100%)	10/10 (100%)	16/16 (100%)	23/24 (96%)	17/17 (100%)	10/10 (100%)	2/2 (100%)
**Prostate**	8 (67%)	5 (46%)	3 (60%)	9 (90%)	14 (88%)	20 (83%)	10 (59%)	8 (80%)	1 (50%)
**PLN**	4 (33%)	4 (36%)	1 (20%)	2 (20%)	3 (19%)	4 (17%)	7 (41%)	2 (20%)	1 (50%)
**Other sites**	8 (67%)	5 (46%)	2 (40%)	3 (30%)	2 (13%)	9 (38%)	10 (59%)	6 (60%)	2 (100%)

DR, detection rate; ISUP, International Society of Urological Pathology; PLN, pelvic lymph nodes; PSAdt, prostate-specific antigen doubling time; RP, radical prostatectomy (± radiotherapy); RT, radiotherapy.
